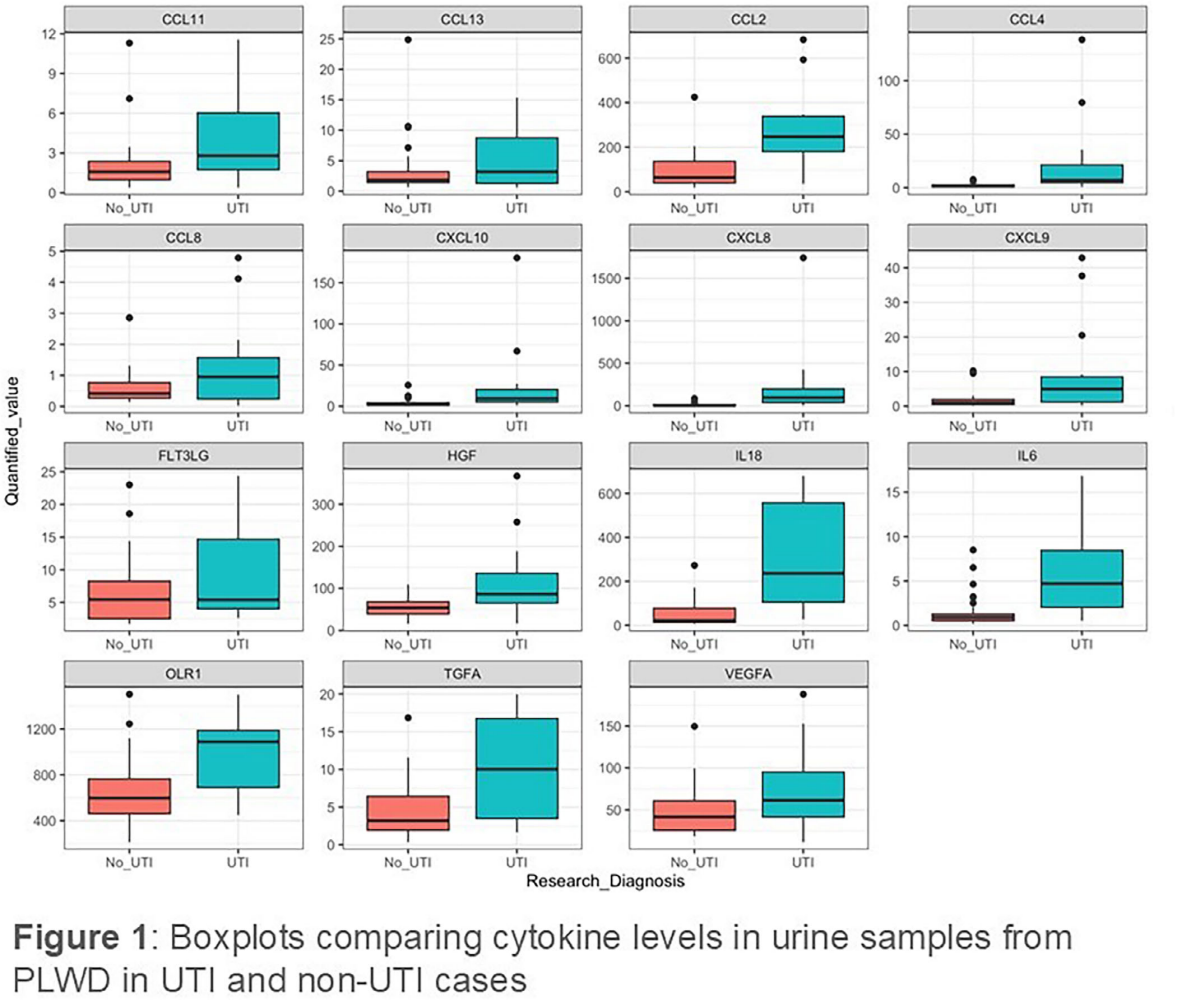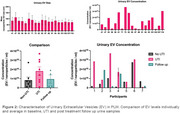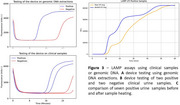# Advancing Early Detection of Urinary Tract Infection in People Living with Dementia

**DOI:** 10.1002/alz70858_103245

**Published:** 2025-12-25

**Authors:** Martin Tran, Thomas Adam, Raphaella Jackson, Rory Cave, Alexander Webb, Richard Kelwick, Michael Crone, Sophie Horrocks, Matthew Harrison, Kirsten Jensen, Paul Freemont

**Affiliations:** ^1^ UK Dementia Research Institute Centre for Care Research and Technology, London, England, United Kingdom; ^2^ Imperial College London, London, England, United Kingdom; ^3^ UK Dementia Research Institute, Care Research and Technology Centre, Imperial College London, London, United Kingdom; ^4^ Helix Centre, Institute of Global Health Innovation, Imperial College London, London, United Kingdom; ^5^ UK Dementia Research Institute Centre for Care Research and TechnologyUK Dementia Research Institute Centre for Care Research and Technology, London, England, United Kingdom

## Abstract

**Background:**

Approximately 25% of hospital beds in the UK are occupied by people living with dementia (PLWD), with up to 20% of these admissions due to preventable acute conditions like infections. Urinary tract infections (UTIs) disproportionately affect PLWD, who often struggle to communicate their symptoms or present atypically. This delays diagnosis, increasing the risk of hospitalisation, sepsis, cognitive decline, and mortality. There is, therefore, a pressing need for improved diagnostic methods to detect UTIs early in PLWD.

**Methods:**

A multi‐omics approach was employed to investigate urinary biomarkers for early UTI detection in PLWD. Longitudinal urine samples were collected from PLWD and analysed for inflammatory markers using OLINK inflammatory panels, focusing on cytokine profiles. Urinary extracellular vesicles (EVs), which are promising UTI biomarkers, were also characterised using nanoflow cytometry. Furthermore, we are sequencing the urinary microbiome to examine microbial dynamics over time whilst also focussing on sequencing *Escherichia coli* genomes isolated from urine samples, to understand their contributions to UTIs. To support future research, urine samples and bacterial isolates were stocked and stored.

Alongside these efforts, we have developed a point‐of‐care (PoC) device that incorporates isothermal amplification for rapid UTI detection. The device uses a heating element for bacterial DNA amplification in urine and an optical detection element to measure fluorescence from DNA dyes.

**Results:**

Our biomarker studies highlight potential inflammatory mediators (figure 1) and elevated EV‐associated markers for early UTI detection (figure 2). Genomic DNA was used to validate the PoC device's accuracy in predicting the presence of *E. coli*. Preliminary experiments observed an earlier fluorescent response from positive DNA extractions compared to negative ones. Whilst clinical sample contaminants reduced assay sensitivity and specificity, heat‐treating samples beneficially increased the levels of detectable cell‐free DNA thereby improving assay performance (figure 3).

**Conclusions:**

The identification of urinary biomarkers and the development of a PoC device offer promising avenues for improving UTI diagnosis in PLWD. Reliable early detection could reduce hospital admissions, prevent severe complications and improve overall outcomes for this vulnerable population. Future work will focus on refining biomarker analyses and optimising the PoC design to enhance its diagnostic accuracy and usability.